# Midazolam Alters Acid-Base Status Less than Azaperone during the Capture and Transport of Southern White Rhinoceroses (*Ceratotherium simum simum*)

**DOI:** 10.3390/ani10081323

**Published:** 2020-07-31

**Authors:** Friederike Pohlin, Peter Buss, Emma H. Hooijberg, Leith C. R. Meyer

**Affiliations:** 1 Research Institute of Wildlife Ecology, Department of Interdisciplinary Life Sciences, University of Veterinary Medicine Vienna, 1160 Vienna, Austria; 2 Centre for Veterinary Wildlife Studies, Faculty of Veterinary Science, Onderstepoort Campus, University of Pretoria, 0110 Pretoria, Gauteng, South Africa; peterb@sanparks.org (P.B.); emma.hooijberg@up.ac.za (E.H.H.); leith.meyer@up.ac.za (L.C.R.M.); 3 Department of Paraclinical Sciences, Faculty of Veterinary Science, Onderstepoort Campus, University of Pretoria, 0110 Pretoria, Gauteng, South Africa; 4 Veterinary Wildlife Services: Kruger National Park, South African National Parks, 1350 Skukuza, Mpumalanga, South Africa; 5 Department of Companion Animal Clinical Studies, Faculty of Veterinary Science, Onderstepoort Campus, University of Pretoria, 0110 Pretoria, Gauteng, South Africa

**Keywords:** acidosis, azaperone, midazolam, rhinoceros, translocation, Henderson-Hasselbalch, Stewart’s approach, wildlife

## Abstract

**Simple Summary:**

Capture and transport are important tools for rhinoceros conservation but are associated with morbidity and even mortality. Changes in acid-base status represent major life-threatening risks during rhinoceros capture and transport. In order to reduce these risks it is important to understand the nature of, and mechanisms contributing to, these acid-base changes. Usually, the tranquilizer azaperone is combined with the immobilizing drug etorphine for rhinoceros capture and is also administered as a tranquilizer during transport. In this study we describe changes in acid-base status during rhinoceros capture and transport and determine whether these changes can be reduced by administering the sedative midazolam, instead of azaperone. Twenty-three wild white rhinoceros bulls were captured with either etorphine-azaperone or etorphine-midazolam. During a 280 km road-transport, either azaperone or midazolam was re-administered every 2 h. All rhinoceroses experienced an increased acidity (low pH) in the blood (acidemia) during capture. Rhinoceroses captured with midazolam suffered less from acidemia than rhinoceroses administered azaperone. In all animals, recovery from the acidemia occurred rapidly after capture and pH remained within normal limits throughout transport. We show that using midazolam instead of azaperone, with the etorphine, may help reduce the risks associated with rhinoceros capture and thus, improve animal welfare during transportation operations.

**Abstract:**

Acidemia represents a major life-threatening factor during rhinoceros capture. The acid-base status during rhinoceros transport is unknown. The purpose of this study was to describe changes in acid-base status during rhinoceros capture and transport and compare these changes between rhinoceroses sedated with azaperone or midazolam. Twenty-three wild white rhinoceros bulls were road-transported 280 km for reasons unrelated to this study. Rhinoceroses were captured with etorphine-azaperone (Group A) or etorphine-midazolam (Group M). During transport, azaperone (Group A) or midazolam (Group M) was re-administered every 2 h and venous blood collected. Changes in blood pH and associated variables were compared over time and between groups using a general linear mixed model. Rhinoceroses of both groups experienced a respiratory and metabolic acidosis during capture (pH 7.109 ± 0.099 and 7.196 ± 0.111 for Group A and Group M, respectively) that was quickly compensated for by the start of transport (pH 7.441 ± 0.035 and 7.430 ± 0.057) and remained stable throughout the journey. Rhinoceroses from Group M showed a smaller decrease in pH and associated variables at capture than rhinoceroses from Group A (*p* = 0.012). The use of midazolam instead of azaperone could therefore improve the success of rhinoceros capture and thus, contribute to the outcome of important conservation translocations.

## 1. Introduction

The southern white rhinoceros (*Ceratotherium simum simum*) is listed as “near threatened” by the International Union for Conservation of Nature (IUCN) Red List of Threatened Species [1]. Rhinoceroses have faced many different threats over the past decades. Habitat loss and fragmentation as well as climate change (droughts) are important factors, but illegal hunting (poaching) for the illegal international rhinoceros horn trade is currently their primary threat [2]. South Africa is host to around 86% of the African white rhinoceros population, the largest of which is found within the Kruger National Park, which has been severely affected by poaching since 2007 [3]. Various conservation strategies have been implemented to combat rhinoceros poaching and ensure long-term survival of viable and valued African rhinoceros populations [4]. Translocation plays a central role in these strategies and is widely used to reinforce declining populations, restore extirpated populations or move animals away from high risk poaching areas [5]. Translocation is defined as the deliberate human-mediated movement of living organisms from one location to another [6] and typically involves capture, transport and release [7]. Despite the widespread use and importance of this practice, and improvements over the years, rhinoceros translocations often result in morbidity and even mortality [8].

Changes in acid-base status might contribute to a negative outcome in rhinoceros translocations. As metabolic reactions can only occur efficiently over a narrow pH range, the body regulates blood pH within very narrow limits (7.35–7.45) [9]. Even small changes in acid-base balance can severely affect physiological and biochemical processes and have potentially fatal consequences [10]. Capture, through immobilization with etorphine-based drug combinations, is known to cause life-threatening respiratory and metabolic acidosis in rhinoceroses [11,12]. The acid-base status during transport is unknown and remains to be investigated. In order to improve translocation outcome, it is important to understand the pathophysiology of acid-base changes caused by capture and transport and investigate drug combinations that could reduce these changes.

There are two different methods that are commonly used to describe acid-base status: the traditional Henderson-Hasselbalch bicarbonate based and the Stewart’s quantitative strong ion difference based approach [9]. To date only the Henderson-Hasselbalch approach has been applied in rhinoceroses [11,13,14]. The Henderson-Hasselbalch approach assumes that the ratio of carbonic acid and bicarbonate determine hydrogen ion concentration and thus pH [15,16]. The limitation of this approach is that accuracy can only be assumed if plasma protein and electrolyte concentrations are within normal limits [17]. White rhinoceroses captured with etorphine experience significant physiological disturbances resulting from the effects of the potent opioid combined with a fight or flight response [18,19]. These disturbances include hypoxemia, hypercapnia, tachycardia and systemic hypertension [18]. Therefore, protein and electrolyte concentrations are unlikely to be within normal limits during the capture of these animals. The Stewart’s approach includes these variables and explains how alterations in plasma protein and phosphate concentrations, as well as changes in the concentration of strong ions such as sodium and chloride, affect pH [17]. Thus, the Stewart’s approach may be better suited to comprehensively assess acid-base changes during rhinoceros capture and transport.

By producing peripheral vasodilation, the butyrophenone azaperone is routinely combined with the etorphine to reduce the potent opioid’s hypertensive effects during capture and as a tranquilizer during transport [20,21]. However, rhinoceroses still develop muscular rigidity, tremors, respiratory depression and respiratory and metabolic acidosis with this combination [11,12]. It has recently been proposed that midazolam is initially co-administered with the etorphine, instead of azaperone, and then used as a sedative during transport [22]. Midazolam is a benzodiazepine that enhances the effects of the neurotransmitter gamma-aminobutyric acid (GABA) at the GABA_A_ receptor resulting in skeletal muscle relaxation, anxiolysis and sedation [23]. Combining midazolam with etorphine could reduce muscular rigidity and the fight or flight response, thereby reducing oxygen consumption, anaerobic metabolism and lactic acidosis [22]. Relaxation of thoracic muscles could further reduce respiratory depression.

The aim of this study was to (1) investigate changes in acid-base status during the capture and transport of white rhinoceroses using the traditional Henderson-Hasselbalch and the Stewart’s quantitative strong ion difference based approach, and (2) to determine whether these changes can be reduced by using midazolam, instead of azaperone, for the capture and transport of wild white rhinoceroses. We hypothesized that (1) blood pH would be lowest in immobilized rhinoceroses during capture and gradually increase during transport and (2) that midazolam would cause fewer changes in the rhinoceroses’ acid-base status than azaperone.

## 2. Material and Methods

A total of 23 sub-adult white rhinoceros bulls were immobilized and road-transported 280 km within the Kruger National Park, for management purposes unrelated to this study. Four animals (three on one translocation) were captured from the wild and transported at a time, resulting in six translocation events over three successive weeks. All procedures were performed according to the Standard Operating Procedure for the Capture, Transportation and Maintenance in Holding Facilities of Wildlife approved by South African National Parks (SANParks) Animal Use and Care Committee (AUCC) (reference number 002/17). The University of Pretoria Animal Ethics and Research Committee (protocol V067-17) and the SANParks AUCC (protocol 009/17) approved the inclusion of these rhinoceroses in this study.

### 2.1. Capture

Rhinoceroses were located by direct observation and drugs were delivered remotely from a helicopter intramuscularly (IM) into the gluteal muscle using 3.0 mL plastic darts with a 60 mm uncollared needle (Dan-Inject; International S.A., Skukuza, South Africa). Two immobilizing-drug protocols were administered alternately: (1) etorphine (etorphine hydrochloride 9.8 mg/mL, Captivon; Wildlife Pharmaceuticals, Karino, South Africa) combined with azaperone (azaperone tartrate 50 mg/mL, Wildlife Pharmaceuticals) (Group A, *n* = 11) and, (2) etorphine combined with midazolam (midazolam hydrochloride 50 mg/mL, Dazonil; Wildlife Pharmaceuticals) (Group M, *n* = 12). Etorphine doses were based on standardized estimated weight categories: 1250–1500 kg = 3 mg; 1500–1750 = 3.5 mg; 1750–2000 = 4 mg, aiming to administer 2 µg/kg. The rhinoceroses’ weight was estimated based on animal size and body condition. Azaperone or midazolam was administered at five times the etorphine dose in mg. These doses have been used in clinical practice and deemed to be optimally effective as “opioid-synergists” in white rhinoceroses [20]. Time from darting to recumbency was recorded as the induction time. Once immobilized, rhinoceroses were approached quietly, positioned in lateral recumbency and blindfolded to reduce stimuli during handling. A blood sample was immediately collected from the immobilized rhinoceroses (time capture sample = TC) from the cephalic vein and a temperature logger (iButton DS1921H-F5# thermochron, iButtonLink, Whitewater, WI, USA) was inserted into the animals’ rectum to record the body temperature during transport. The temperature logger was attached to a string, which was tied to the animals’ tail in order to allow for easy removal after transport. The skin of the right ear was aseptically prepared and a 16 gauge 20-cm over-the-wire intravenous catheter (Arrow; Teleflex, Morrisville, NC, USA) inserted into an auricular vein and sutured to the skin to allow for serial blood sample collection during transport. Throughout this 30-min procedure, heart rate, respiratory rate, and body temperature were monitored, and oxygen provided by nasal insufflation at 10 L/min from a portable oxygen tank. Once the catheter was in place, butorphanol (butorphanol tartrate 50 mg/mL, Wildlife Pharmaceuticals) (5 mg per mg etorphine) was administered intravenously (IV) to partially antagonize the immobilizing effects of the etorphine and enable the rhinoceroses to walk into a transport crate [14]. Once in the crate, an IV bolus of diprenorphine (diprenorphine hydrochloride 12 mg/mL, Activon; Wildlife Pharmaceuticals) (3 mg per mg etorphine) was administered to further antagonize residual immobilizing, but not sedative, effects of the etorphine [24].

### 2.2. Transport

Rhinoceroses were transported on trucks in International Air Transport Association (IATA, Montreal, QC, Canada) approved crates, following the practical guidelines for transport of live wild animals [25] and rhinoceroses [5,26]. Transport started once four rhinoceroses (three on one translocation) had been captured and loaded into the transport crates (time 0 h start transport sample = T0). Azaperone (Group A) or midazolam (Group M) was re-administered IM via hand-injection into the nuchal hump at a standard dose of 25 times the etorphine dose in mg at the start of transport (T0) and at two (T2) and four (T4) hours of transport. During these drug-administration stops, blood samples were also collected from each rhinoceros using the auricular catheter. The destination was reached after six hours of transport (time 6 h transport sample = T6). A final blood sample was collected, the auricular catheter and rectal temperature logger removed, and naltrexone (naltrexone hydrochloride 50 mg/mL, Trexonil; Wildlife Pharmaceuticals) (20 mg per mg etorphine) administered IV to antagonize residual etorphine effects and the rhinoceroses were then released back into the wild.

### 2.3. Sample Collection and Analysis

Blood was collected into lithium-heparinized tubes (BD Vacutainer^®^; Becton and Dickinson, Oxford, UK) and analyzed immediately using a portable point-of-care blood gas analyzer with test cards (EPOC^®^ Portable analyzer system + EPOC^®^ BGEM test cards, Kyron Laboratories, Johannesburg, South Africa). The device measured pH, partial pressure of carbon dioxide (PCO_2_), concentrations of sodium (Na^+^), potassium (K^+^), ionized calcium (iCa^++^), chloride (Cl^−^), glucose, and lactate; and calculated bicarbonate (HCO_3_^−^), base excess (BE), and anion gap (AG) using the Henderson-Hasselbalch equation [15,16]. Blood samples collected into serum tubes (BD Vacutainer^®^; Becton and Dickinson) were stored in a cooler box with ice packs, centrifuged within 24 h of sample collection and stored at −80 °C for 1 month until analyzed in the clinical pathology laboratory of the Faculty of Veterinary Science, University of Pretoria. Concentrations of selected serum clinical chemistry analytes were measured using a Cobas Integra 400 Plus automated biochemistry analyzer (Roche Diagnostics Ltd., Rotkreuz, Switzerland) and commercially available kits (Roche Diagnostics Ltd.). Total Magnesium (Mg), inorganic phosphate (Pi), albumin and globulin (calculated as total protein minus albumin) were measured at all time points. Urea and creatinine were only measured at TC, T0 and T6.

### 2.4. Calculated Variables

Quantitative analysis of acid-base status was assessed using the Stewart’s approach to acid-base chemistry [27] simplified by Constable (1997) [28]. Measured strong ion difference (SIDm), total non-volatile weak acids (Atot) and strong ion gap (SIG) were estimated using the following formulas, derived for horses [28]:

SIDm (mmol/L) = (Na^+^ + K^+^ + iCa^++^ + Mg) − (Cl^−^ + lactate)

Atot (mmol/L) = 0.225 × albumin (g/L) + 0.14 × globulin (g/L) + 1.8 × Pi (mmol/L)

SIG (mmol/L) = Atot/(1 + 10^pKa-pH^) − AG

The value for the pKa of weak acids is unknown for rhinoceros plasma, but was experimentally determined as 6.65 in the horse, the closest domesticated relative to the rhinoceros [28], and so a pKa of 6.65 was used in our study. Plasma osmolality was calculated as [29,30]:

Osmolality (mOsm/kg) = 2 × (Na^+^ + K^+^) + glucose (mmol/L) + urea (mmol/L)

Interpretation of acid-base status was performed using the Henderson-Hasselbalch and Stewart’s approach. Reference intervals for white rhinoceroses, using exactly the same analytical methods and sample types as described here, are only available for selected clinical chemistry measurands [31]. Other studies report reference values gathered using different methods or samples, so data are not directly comparable, but were still used to assist with interpretation of our findings [32,33,34,35]. Information from horses was used if rhinoceros reference values were not available (e.g., for Atot) [36,37].

### 2.5. Statistical Analysis

All statistical analyses were performed with the software R version 3.6.1 (R Foundation for Statistical Computing, Vienna, Austria) [38]. Data were assessed for normality by calculating descriptive statistics and plotting of histograms. Mean and standard deviations (SD) were calculated for each analyte per sample time point and group and interval plots were generated for descriptive purposes. Changes over time and between groups for variables of interest were compared using a general linear mixed model (fixed factors: time and group; random factors: rhinoceros; interactions: time × group). Start of transport (T0) and Group A were used as reference category to differentiate the effects of capture (TC to T0) from the effects of transport (T0 to T6) and because azaperone is the drug that is currently most commonly added to etorphine for rhinoceros capture and transport. Induction times (period between darting and immobilization) were compared between groups using a Mann-Whitney-Test. A *p*-value <0.05 was considered significant.

## 3. Results

All rhinoceroses survived capture and transport. Ambient temperatures ranged from 16.9 ± 1.2 (mean ± SD) °C to 27.7 ± 4.3 °C during the translocations. Rhinoceroses were weighed while in the transport crate and the drug doses used for the immobilization and sedation during transport recalculated on a per kilogram basis. The animal’s actual weight ranged from 1155 to 2046 (1547 ± 238) kg, which was slightly less than estimated. Thus, etorphine and azaperone had been administered at 2.49 ± 0.38 and 12.27 ± 2.09 µg/kg, respectively, in Group A, and at 2.58 ± 0.37 and 12.07 ± 1.86 µg/kg, respectively, in Group M. During transport azaperone had been administered at 62.38 ± 9.54 µg/kg, and midazolam at 64.61 ± 9.28 µg/kg. The induction time did not differ between the groups (*p* = 0.717) and was 7:37 ± 2:57 min (Group A) and 7:55 ± 2:54 min (Group M). As we could only capture one rhinoceros at a time, the individuals captured at first had to wait in the transport crates until all four animals had been caught and transport was started. This limitation resulted in a time-lag from TC to T0 between individuals, which was 189 ± 84 min in Group A and 117 ± 73 min in Group M (*p* = 0.031). Of all rectal temperature loggers, only one animal from Group A lost its logger during transport. Body temperature data from this animal was therefore not included in the study. In the remaining rhinoceroses, body temperature at TC was 38.0 ± 0.6 °C in Group A and 38.1 ± 0.5 °C in Group M. Body temperature did not change significantly from TC to T0, but decreased to 37.2 ± 0.8 °C and 37.3 ± 0.8 °C, at T6 in Group A and M, respectively (*p* = 0.01). There were no significant differences in body temperature between the groups and body temperature remained within normal ranges for anesthetized rhinoceroses (37 °C–39 °C [21]) in all animals.

Descriptive analysis of results is provided in Table 1 and Table S1. Mean ± SD of variables used in the interpretation of acid-base status are described in Table 1. Mean ± SD of measured serum clinical chemistry analyte concentrations used to calculate dependent variables, or aid in the interpretation of results, are described in Table S1.

We also provide model-based analysis including the interaction effects of time and group. Coefficient estimates and corresponding standard errors and *p*-values are presented in Table 2. Across all parameters, we found a strong and significant effect of time with the greatest changes in blood acid-base status occurring between TC and T0. Briefly, blood pH, HCO_3_^−^, BE, SIDm, and SIG increased from TC to T0 (*p* < 0.001 all variables), and venous PCO2, lactate, AG, Atot and osmolality decreased from TC to T0 (*p* < 0.001 all variables) (mean ± SD for both groups are listed in Table 1).

Our model results show no significant main effects of midazolam on any of the acid-based variables. However, with respect to the interaction effect between time and Group, we found a number of significant interaction effects of TC and midazolam. There was a positive and significant interaction effect of TC and midazolam for pH (*p* = 0.012), BE (*p* = 0.027) and SIG (*p* = 0.002) and a negative and significant interaction effect of TC and midazolam for lactate (*p* = 0.002), AG (*p* = 0.003) and osmolality (*p* = 0.011). Thus, rhinoceroses from Group M showed a smaller increase in pH, BE and SIG and smaller decrease in lactate, AG and osmolality from TC to T0 than rhinoceroses from Group A.

## 4. Discussion

The initial blood pH, in the immobilized animals at TC, indicated a pronounced acidemia (normal arterial blood pH range 7.346 to 7.431, reported from unrestrained standing rhinoceroses [33]) in the rhinoceroses of both groups. Elevated PCO_2_ at this time caused a respiratory acidosis, while increased lactate concentrations and associated changes in AG, SIG and SID, together with a mild increase in Atot, indicated a simultaneously occurring metabolic acidosis. There was a strong effect of time from TC to T0. By T0, these acid-base imbalances had already normalized and remained within normal limits throughout the journey (T0 to T6), where there was no significant main effect of time. The interaction of Group M and TC suggested that rhinoceroses captured with etorphine-midazolam suffered less from lactic acidosis and associated electrolyte shifts than rhinoceroses captured with etorphine-azaperone. There were no statistically significant interaction effects of midazolam and the other time points.

### 4.1. Changes in Acid-Base Status during Capture and Transport

Respiratory acidosis is a common finding in rhinoceroses immobilized with the potent opioid etorphine [12]. Immediately following capture, venous PCO_2_ values were greatly elevated in our rhinoceroses (normal 44.4–53.7 mmHg [33]). Increases in PCO_2_ are indicative of impaired alveolar ventilation and are thought to be the result of respiratory neuronal depression [39] and thoracic muscular rigidity [20] caused by the etorphine. Ventilation-perfusion mismatching and shunting during prolonged lateral recumbency likely contributed to the hypercarbia in our animals [40] together with increased carbon dioxide production from etorphine-induced hyper-metabolism [19]. By the start of transport venous PCO_2_ had decreased to normal values [33], which were maintained throughout the journey. Partial reversal of the etorphine by administering the opioid agonist-antagonists butorphanol and diprenorphine [24] and the change in body position, from lateral recumbency to standing, rapidly improved alveolar ventilation [13] and reduced hyper-metabolism [19]. These ventilatory changes likely had the greatest compensatory effects on the pH from TC to T0 but increases in HCO_3_^−^ and BE at this time indicated that a blood intracellular chemical buffer response had also taken place [41]. Venous HCO_3_^−^ and BE remained constant throughout transport (T0–T6), within a similar range as reported by Citino & Bush (2007) [33] in arterial blood in unrestrained standing white rhinoceroses (27.3–32.2 mm/L and 1.9–5.9 for HCO_3_^−^ and BE, respectively).

Compared to reference values from ground immobilized white rhinoceroses (4.6 mmol/L) [35] and resting horses (<2 mmol/L) [37], blood lactate concentrations were markedly elevated at TC. This hyperlactatemia was reflected in a higher AG and negative SIG at TC compared to T0 and during transport (T0–T6) indicating an increase in unmeasured (strong) anions such as lactate [37]. Measured electrolyte concentrations (Na^+^, Cl^−^, K^+^, iCa^++^, Pi, Mg) remained largely within accepted published ranges for rhinoceroses [31,32,34]. Therefore, changes in lactate concentration likely also caused the concurrent changes in SIDm [42]. Hyperlactatemia is the result of an increased lactate production during oxygen debt and leads to hydrogen ion generation and acidosis [43]. An increase in lactate production during capture was not unexpected, as rhinoceroses were darted from a helicopter and experienced high levels of muscular activity from exertion, and drug-induced increase in oxygen consumption and hypoxia prior to and during immobilization [12]. Recovery from the hyperlactatemia and associated lactic acidosis had occurred by the start of transport indicating that most of the excess lactate had been rapidly utilized by the muscles and recycled by the liver and other organs [43]. Generated hydrogen ions were likely buffered by extracellular and intracellular chemical buffer systems [41].

Plasma proteins and phosphates are weak acids that are not fully dissociated at physiological pH and are able to buffer hydrogen ions [42]. In the Stewart’s approach, Atot represents the total plasma concentration of non-volatile weak acids [28]. At TC, Atot was significantly higher than during transport (T0–T6) and compared to equine reference intervals (10.1–15.5 mEq/L) [36]. Increased Atot results in a pH decrease and is often a consequence of hemoconcentration [37]. Rhinoceroses, particularly if captured from the wild, exhibit a fight or flight response causing severe systemic arterial hypertension after darting with etorphine-based drug combinations [18,19]. The associated increase in hydrostatic pressure likely led to a shift of plasma from the intravascular compartment into interstitial spaces causing hemoconcentration and a total weak acid acidosis [44]. The mildly higher plasma osmolality at capture, compared to transport, was likely caused by an increase in glucose concentrations associated with a stress response to capture [45]. A concurrent loss in total body water is unlikely, as Na^+^, urea and creatinine concentrations did not change from TC to T0.

### 4.2. Difference in Acid-Base Status between the Groups

Rhinoceroses captured with etorphine-midazolam (Group M) suffered less from lactic acidosis and changes in associated calculated variables (AG, BE and SIG) than rhinoceroses captured with etorphine-azaperone (Group A). Due to the midazolam’s muscle relaxant effects, these rhinoceroses likely experienced less muscular rigidity resulting in a less severe hyperlactatemia. The lower osmolality at TC in the rhinoceroses of this group, was likely the result of smaller electrolyte shifts associated with smaller pH changes [46]. A tendency towards lower PCO_2_ in Group M was noted, indicating that rhinoceroses captured with etorphine-midazolam may have also ventilated better.

There were no differences between Group A and M during transport, where acid-base measurements had returned to normal limits, at the doses and administration intervals used in this study. Compared to standard doses used for equine sedation (0.01–0.2 mg/kg) [47], the midazolam doses used in our rhinoceroses were relatively low. Previous reports suggest a total midazolam dose as little as 10 mg is sufficient for the transport of an adult rhinoceros [20]. For practical reasons, we administered midazolam at the same dose and time interval as recommended for azaperone during rhinoceros transport [20]. Future studies could investigate single and multiple IM dose pharmacokinetics of midazolam in rhinoceroses and establish scientifically proven recommendations for the use of this drug in rhinoceros capture and transport. In these studies, arterial blood samples should also be collected, and blood oxygenation evaluated in order to detect any respiratory benefits of using midazolam over azaperone. Moreover, other potential benefits of using midazolam, instead of azaperone, such as greater anxiolytic and stress reducing effects, should be investigated during rhinoceros capture and transport.

### 4.3. Limitations

Due to the rhinoceroses’ conscious state in the crates and their wild nature, arterial anaerobic blood sample collection was not possible in this study. However, venous pH, PCO_2_ and HCO_3_^−^ are known to be reliable indicators of acid-base status and are therefore a good surrogate where it is not possible to collect arterial samples [48].

Due to logistical factors, we transported rhinoceroses on six different translocation events. Different environmental conditions during these events might have influenced our results and cannot be excluded. Moreover, because we could not capture more than one rhinoceros at a time, this resulted in a time lag from TC to T0 between individuals, which represents another limitation to the study in addition to the small sample size.

For practical reasons, we collected venous blood samples from two different venipuncture sites, the cephalic (TC) and auricular vein (T0–T6). The choice of venipuncture site has recently been shown to not influence rhinoceros hematological and biochemical variables as long as the same anticoagulant is used [49]. We believe that these limitations only caused minimal alterations in our rhinoceroses.

## 5. Conclusions

Repetitive blood sample collection within individuals has allowed us to detect the trend of acid-base variables over time and identify major changes in acid-base status during rhinoceros capture and transport. Capture caused the greatest imbalance to the acid-base status in all rhinoceroses by inducing respiratory acidosis combined with a metabolic (lactic- and non-volatile weak acid) acidosis. This acidosis was quickly compensated for by the start of transport and blood pH remained stable throughout transport. These results show that, from an acid-base perspective, capture is the most critical risk period of translocation, and is when life-threatening acid-base changes occur, and that transport of six hours does not further challenge rhinoceros welfare with acid-base alterations. Future studies aiming at improving acid-base status during rhinoceros translocation should therefore focus on the immobilized animal during capture.

Secondly, by comparing two drug protocols, this study has allowed us to determine that there are significant differences in acid-base status resulting from the use of midazolam compared to azaperone for rhinoceros capture and transport. Midazolam was able to limit the metabolic (lactic) acidosis seen at capture and therefore represents a promising alternative to azaperone when combined with etorphine for wild white rhinoceros immobilization.

## Figures and Tables

**Table 1:** Mean ± standard deviation for pH, venous partial pressure of carbon dioxide (PCO_2_), bicarbonate (HCO_3_^−^), base excess (BE), anion gap (AG), lactate, measured strong ion difference (SIDm), non-volatile weak acids (Atot), strong ion gap (SIG) and plasma osmolality in rhinoceroses captured and transported with either azaperone (Group A) or midazolam (Group M) as a sedative. Time: capture (TC), start of transport (T0), and two (T2), four (T4) and six (T6) hours of transport.

Variable (Unit)	Group	Time				
		TC	T0	T2	T4	T6
pH	A	7.109 ± 0.099	7.441 ± 0.035	7.443 ± 0.04	7.479 ± 0.055	7.474 ± 0.068
	M	7.196 ± 0.111	7.430 ± 0.057	7.463 ± 0.037	7.469 ± 0.046	7.474 ± 0.056
Traditional (Henderson-Hasselbalch) blood acid-base variables						
PCO_2_	A	73.3 ± 9.9	49.6 ± 0.1	51.2 ± 6.6	46.1 ±7.1	45.9 ± 8.9
(mmHg)	M	65.4 ± 10.3	48.7 ± 7.4	47.6 ± 6.6	46.6 ± 6.9	46.7 ± 8.0
HCO_3_^−^	A	23.7 ± 5.3	33.9 ± 2.0	34.8 ± 2.1	33.9 ± 1.7	33.1 ± 2.2
(mmol/L)	M	25.9 ± 5.8	32.5 ± 4.6	33.8 ± 3.0	33.6 ± 2.6	33.9 ± 3.0
BE	A	−5.8 ± 6.7	9.7 ± 1.8	10.7 ± 2.3	10.4 ± 1.2	9.5 ± 2.0
(mmol/L)	M	−2.2 ± 7.3	8.3 ± 5.1	10.0 ± 2.9	9.9 ± 2.4	11.3 ± 4.5
AG	A	21 ± 5	12 ± 1	11 ± 2	12 ± 2	13 ± 2
(mmol/L)	M	17 ± 5	13 ± 4	12 ± 2	13 ±2	13 ± 2
Lactate	A	12.04 ± 4.21	2.38 ± 0.93	2.01 ± 0.93	2.41 ± 1.57	2.54 ± 1.67
(mmol/L)	M	8.82 ± 5.07	3.77 ± 3.23	2.32 ± 1.17	2.91 ± 1.35	2.71 ± 0.91
Quantitative (Stewart’s) blood acid-base variables						
SIDm	A	35.2 ± 5.4	46.0 ± 1.7	46.1 ± 1.5	46.1 ± 1.0	45.9 ± 2.4
(mmol/L)	M	36.7 ± 5.8	44.4 ± 4.5	45.7 ± 2.7	45.5 ± 2.6	46.7 ± 2.2
Atot	A	17.5 ± 0.6	15.5 ± 1.3	15.9 ± 1.3	15.7 ± 1.3	15.4 ± 1.3
(mmol/L)	M	17.2 ± 0.7	14.9 ± 0.5	15.3 ± 0.7	15.1 ± 0.5	15.1 ± 0.5
SIG	A	−8.0 ± 5.4	1.1 ± 1.9	2.7 ± 1.7	1.3 ± 2.2	0.4 ± 1.7
(mmol/L)	M	−3.6 ± 5.8	−0.5 ± 3.5	2.1 ± 4.0	0.48 ± 1.79	1.9 ± 3.9
Osmolality	A	291.1 ± 9.4	286.2 ± 8.9			286.7 ± 7.2
(mOsm/kg)	M	289.4 ± 8.0	288.8 ± 7.7			286.8 ± 6.7

**Table 2:** Coefficient estimates (standard errors) and *p*-values for time and group on pH, venous partial pressure of carbon dioxide (PCO_2_), bicarbonate (HCO_3_^−^), base excess (BE), anion gap (AG), lactate, measured strong ion difference (SIDm), non-volatile weak acids (Atot), strong ion gap (SIG) and plasma osmolality. Time: capture (TC), start of transport (T0), and two (T2), four (T4) and six (T6) hours of transport. Groups: midazolam (Group M), azaperone (Group A). Reference category: Group A Time T0. The star indicates statistical significance (*p* < 0.05).

	pH	PCO_2_ (mmHg)	HCO_3_^−^ (mmol/L)	BE (mmol/L)	AG (mmol/L)	Lactate (mmol/L)	SIDm (mmol/L)	Atot (mmol/L)	SIG (mmol/L)	Osmolality (mOsm/kg)
Group M	−0.011	−0.986	−1.358	−1.462	1.068	1.392	−1.593	−0.527	−1.572	2.502
	(0.027)	(3.300)	(1.486)	(1.748)	(1.243)	(1.070)	(1.423)	(0.390)	(1.475)	(3.314)
	*p* = 0.695	*p* = 0.766	*p* = 0.362	*p* = 0.403	*p* = 0.391	*p* = 0.193	*p* = 0.263	*p* = 0.177	*p* = 0.287	*p* = 0.451
Time TC	−0.332 *	23.645 *	−10.218 *	−15.573 *	8.818 *	9.657 *	−10.845 *	2.068 *	−9.160 *	4.735 *
	(0.028)	(3.188)	(1.391)	(1.661)	(1.208)	(1.025)	(1.357)	(0.222)	(1.404)	(1.176)
	*p* < 0.001	*p* < 0.001	*p* < 0.001	*p* < 0.001	*p* < 0.001	*p* < 0.001	*p* < 0.001	*p* < 0.001	*p* < 0.001	*p* < 0.001
Time T2	0.002	1.573	0.873	0.945	−1.182	−0.372	0.023	0.433	1.559	
	(0.028)	(3.188)	(1.391)	(1.661)	(1.208)	(1.025)	(1.357)	(0.222)	(1.404)	
	*p* = 0.933	*p* = 0.622	*p* = 0.531	*p* = 0.570	*p* = 0.329	*p* = 0.717	*p* = 0.987	*p* = 0.052	*p* = 0.267	
Time T4	0.038	−3.518	0.036	0.664	0.182	0.028	0.069	0.219	0.164	
	(0.028)	(3.188)	(1.391)	(1.661)	(1.208)	(1.025)	(1.357)	(0.222)	(1.404)	
	*p* = 0.169	*p* = 0.270	*p* = 0.980	*p* = 0.690	*p* = 0.881	*p* = 0.979	*p* = 0.960	*p* = 0.325	*p* = 0.908	
Time T6	0.033	−3.782	−0.782	−0.264	0.727	0.072	−0.337	−0.084	−0.670	0.476
	(0.028)	(3.188)	(1.391)	(1.661)	(1.208)	(1.025)	(1.357)	(0.222)	(1.404)	(1.135)
	*p* = 0.241	*p* = 0.236	*p* = 0.574	*p* = 0.874	*p* = 0.548	*p* = 0.945	*p* = 0.804	*p* = 0.706	*p* = 0.634	*p* = 0.675
Group M: Time TC	0.098 *	−6.879	3.577	5.106 *	−5.152 *	−4.610 *	3.085	0.178	6.013 *	−4.075 *
	(0.039)	(4.413)	(1.925)	(2.300)	(1.673)	(1.419)	(1.879)	(0.308)	(1.943)	(1.602)
	*p* = 0.012	*p* = 0.120	*p* = 0.064	*p* = 0.027	*p* = 0.003	*p* = 0.002	*p* = 0.101	*p* = 0.563	*p* = 0.002	*p* = 0.011
Group M: Time T2	0.030	−2.673	0.386	0.763	0.108	−1.083	1.276	−0.099	0.951	
	(0.039)	(4.413)	(1.925)	(2.300)	(1.692)	(1.419)	(1.901)	(0.308)	(1.943)	
	*p* = 0.437	*p* = 0.545	*p* = 0.842	*p* = 0.741	*p* = 0.950	*p* = 0.446	*p* = 0.503	*p* = 0.748	*p* = 0.625	
Group M: Time T4	−0.0001	1.485	1.014	0.936	−0.848	−0.896	1.021	−0.092	0.773	
	(0.039)	(4.413)	(1.925)	(2.300)	(1.673)	(1.419)	(1.879)	(0.308)	(1.943)	
	*p* = 0.998	*p* = 0.737	*p* = 0.599	*p* = 0.684	*p* = 0.613	*p* = 0.528	*p* = 0.587	*p* = 0.766	*p* = 0.691	
Group M: Time T6	0.011	1.873	2.173	3.230	−1.165	−1.123	2.607	0.333	3.078	−2.475
	(0.039)	(4.413)	(1.925)	(2.300)	(1.692)	(1.419)	(1.927)	(0.312)	(1.966)	(1.572)
	*p* = 0.775	*p* = 0.672	*p* = 0.259	*p* = 0.161	*p* = 0.492	*p* = 0.429	*p* = 0.177	*p* = 0.286	*p* = 0.118	*p* = 0.116
Constant	7.441 *	49.636 *	33.891 *	9.745 *	12.182 *	2.381 *	46.029 *	15.457 *	1.119	286.265 *
	(0.020)	(2.384)	(1.074)	(1.263)	(0.898)	(0.773)	(1.028)	(0.282)	(1.066)	(2.393)
	*p* < 0.001	*p* < 0.001	*p* < 0.001	*p* < 0.001	*p* < 0.001	*p =* 0.003	*p* < 0.001	*p* < 0.001	*p* = 0.294	*p* < 0.001
Observations	115	115	115	115	113	115	112	114	114	68
LL	124.934	−377.299	−292.391	−310.037	−269.919	−258.737	−280.524	−118.074	−289.566	−189.428
AIC	−225.868	778.597	608.782	644.074	563.837	541.474	585.048	260.147	603.131	394.857

## Supplementary Materials

The following are available online at https://www.mdpi.com/2076-2615/10/8/1323/s1, Table S1: Mean ± standard deviation for measured clinical chemistry analyte concentrations used to calculate, or interpret, dependent acid-base variables: sodium (Na^+^), potassium (K^+^), chloride (Cl^−^), ionized calcium (iCa^++^), magnesium (Mg), inorganic phosphorus (Pi), albumin, globulin, glucose, urea, and creatinine in rhinoceroses captured and transported with either azaperone (group A) or midazolam (group M). Time: capture (TC), start of transport (T0), and two (T2), four (T4) and six (T6) hours of transport.
