# Supplementary material for: Midazolam Alters Acid-Base Status Less than Azaperone during the Capture and Transport of Southern White Rhinoceroses (Ceratotherium simum simum)
Source: Animals (Basel). 2020 Jul 31;10(8):1323. doi: 10.3390/ani10081323 (PMC7460343; doi:10.3390/ani10081323)
Supplement: Supplementary file 1 [file animals-10-01323-s001.pdf]

animals

MDPI

Supplementary Files: Midazolam Alters Acid-Base Status Less than Azaperone during the Capture and Transport of Southern White Rhinoceroses (*Ceratotherium simum simum*)

Friederike Pohlin [1,2,3,*], Peter Buss [2,4], Emma H. Hooijberg [2,5] and Leith C.R. Meyer [2,3]

Table S1. Mean ± standard deviation for measured clinical chemistry analyte concentrations used to calculate, or interpret, dependent acid-base variables: sodium (Na^+), potassium (K^+), chloride (Cl^-), ionized calcium (iCa^{++}), magnesium (Mg), inorganic phosphorus (Pi), albumin, globulin, glucose, urea, and creatinine in rhinoceroses captured and transported with either azaperone (group A) or midazolam (group M). Time: capture (TC), start of transport (T0), and two (T2), four (T4) and six (T6) hours of transport.

Variable (unit)	Group	Time				
		TC	T0	T2	T4	T6
Na+	A	135 ± 4	134 ± 4	134 ± 4	134 ± 4	135 ± 3
(mmol/L)	M	133 ± 4	135 ± 3	134 ± 3	134 ± 4	135 ± 4
K^+	A	5.0 ± 0.4	4.1 ± 0.4	3.4 ± 0.3	3.1 ± 0.3	3.1 ± 0.3
(mmol/L)	M	4.7 ± 0.5	4.4 ± 0.4	3.6 ± 0.3	3.2 ± 0.3	3.0 ± 0.2
Cl-	A	95 ± 5	92 ± 4	92 ± 4	91 ± 5	92 ± 5
(mmol/L)	M	95 ± 5	93 ± 4	93 ± 5	91 ± 5	91 ± 5
iCa^{++}	A	1.53 ± 0.08	1.40 ± 0.08	1.41 ± 0.08	1.38 ± 0.06	1.40 ± 0.08
(mmol/L)	M	1.47 ± 0.05	1.38 ± 0.04	1.37 ± 0.05	1.36 ± 0.08	1.38 ± 0.08
Mg	A	1.26 ± 0.11	1.01 ± 0.09	0.95 ± 0.08	0.89 ± 0.08	0.84 ± 0.06
(mmol/L)	M	1.25 ± 0.12	1.04 ± 0.08	0.95 ± 0.04	0.88 ± 0.05	0.89 ± 0.13
Pi	A	1.46 ± 0.21	0.99 ± 0.23	0.97 ± 0.22	0.97 ± 0.24	0.92 ± 0.24
(mmol/L)	M	1.42 ± 0.15	1.05 ± 0.24	0.90 ± 0.29	0.85 ± 0.26	0.83 ± 0.30
Albumin	A	26.6 ± 1.5	24.6 ± 1.0	25.6 ± 1.5	25.8 ± 1.1	25.0 ± 0.7
(g/L)	M	27.4 ± 1.4	24.6 ± 1.8	26.2 ± 1.3	26.3 ± 1.5	26.1 ± 1.1
Globulin	A	63.4 ± 7.0	57.9 ± 8.8	59.5 ± 8.6	57.9 ± 8.3	57.6 ± 9.2
(g/L)	M	60.0 ± 4.1	53.4 ± 3.8	5..2 ± 3.6	54.2 ± 2.7	55.1 ± 3.7
Glucose	A	8.5 ± 2.7	6.3 ± 2.3	6.5 ± 1.9	7.1 ± 1.3	7.6 ± 1.1
(mmol/L)	M	10.1 ± 2.8	7.5 ± 2.4	7.2 ± 1.6	8.1 ± 1.8	8.0 ± 1.7
Urea	A	3.31 ± 0.37	3.44 ± 0.40	-	-	3.70 ± 0.41
(mmol/L)	M	3.28 ± 0.51	3.40 ± 0.47	-	-	3.82 ± 0.42
Creatinine	A	145 ± 22	143 ± 24	-	-	139 ± 26
(µmol/L)	M	149 ± 30	150 ± 29	-	-	148 ± 29
